# Kombucha as a Bioactive Functional Beverage: Current Evidence, Production Challenges, and Future Perspectives

**DOI:** 10.3390/ijms27062739

**Published:** 2026-03-17

**Authors:** Antia G. Pereira

**Affiliations:** 1 Nutrition and Food Group (NuFoG), Department of Analytical Chemistry and Food Science, Instituto de Agroecoloxía e Alimentación (IAA), Campus Auga, Universidade de Vigo, 32004 Ourense, Spain; antia.gonzalez.pereira@uvigo.es; 2 Nutrition and Food Group (NuFoG), Galicia Sur Health Research Institute (IIS Galicia Sur), SERGAS-UVIGO, 36213 Vigo, Spain

**Keywords:** fermented tea, microbial consortium, bioactive compounds, fermentation process, functional ingredients, beverage safety

## Abstract

Kombucha is a beverage obtained through the fermentation of tea leaves by a symbiotic culture of bacteria and yeast called SCOBY. This beverage is popularly known for the potential health benefits associated with its consumption, which is the main reason for its commercial expansion over the last century. These potential benefits are linked to the presence of a wide variety of bioactive compounds, notably phenolic compounds and organic acids. This composition varies significantly depending on the fermentation conditions, which in turn modifies the beverage’s bioactive properties (i.e., antioxidant capacity or antimicrobial properties, among others). For this reason, the most recent advances in kombucha production are geared towards achieving standardized production, including strategies for enhancing bioactive content and fortification with functional ingredients. All these advances should satisfy quality control and regulatory compliance. However, despite the growing scientific and commercial interest in kombucha, current knowledge remains fragmented across different disciplines, highlighting the need for an updated and integrative overview of its composition, bioactivity, production variables, and safety aspects. In this review, nutritional, microbiological, and technological perspectives are integrated to provide an updated framework for understanding kombucha as a functional beverage, while also outlining key directions for future research and industrial application.

## 1. Introduction

**Figure 1:**
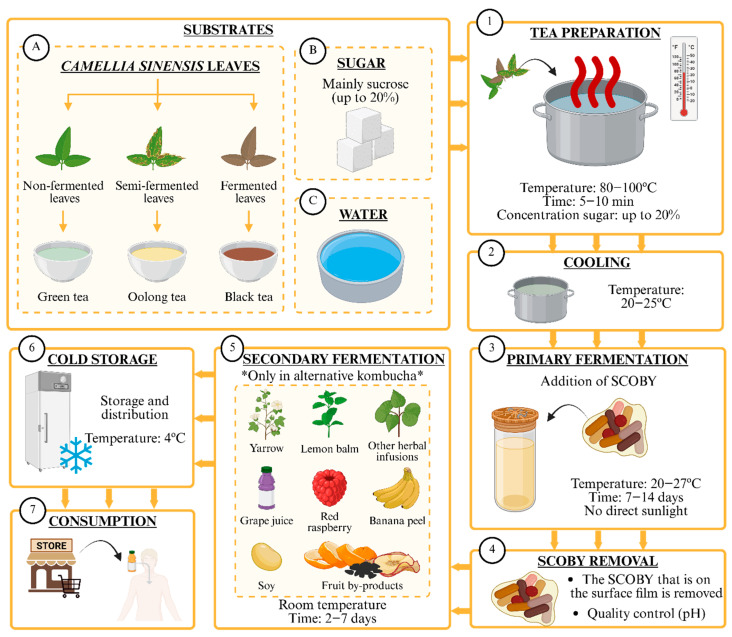
Production of kombucha.

Kombucha is a non-alcoholic or low-alcohol, fermented beverage made from sweetened tea (mainly black and green tea with 5–10% sucrose), and other herbal infusions that can be added during the secondary fermentation step [1]. Following the conventional procedure, black or green tea is infused with sucrose for five minutes and subsequently allowed to cool to ambient temperature (Figure 1). The infusion is then filtered, after which a portion of kombucha from a previous batch is incorporated as the starter culture [2,3]. This starter culture is known as a SCOBY (symbiotic colony of bacteria and yeast) or “mother” [4]. These microorganisms mostly belong to the Acetobacteraceae family (*Acetobacter aceti*, *Acetobacter estunensis*, *Acetobacter pasteurianus*, *Gluconobacter oxydans*, *Komagataeibacter kombuchae*, *Komagataeibacter. rhaeticus*, *and Komagataeibacter xylinus*), *Lactobacillus* sp. as well as osmophilic yeasts (*Brettanomyces*/*Dekkera*, *Candida*, *Saccharomyces*, *Schizosaccharomyces*, *Starmerella* sp., *Torulopsis*, *Pichia* sp., and *Zygosaccharomyces*) [2,3]. The mixture is then covered and allowed to ferment under dark conditions for 10–14 days, during which the product, commonly referred to as “tea fungus,” is formed. This tea fungus is characterized by having a cellulose-like film on the surface of the fermentation medium, a film that will be used as a starter culture in the next production batch [4]. During this period, the product develops its characteristic organoleptic properties, most notably its slightly acidic and refreshing flavor, and a mild natural effervescence resulting from carbon dioxide production during fermentation. Some of the beverage’s sensory properties can be further modified during the secondary fermentation, in which additional ingredients such as fruit juices, plant extracts, or other functional components are added to enhance flavor, aroma, or bioactive content [5,6].

The origin of this beverage dates back to 220 BC in ancient China during the Qin Dynasty, where it was valued for its organoleptic properties and potential health benefits, leading to its designation as “immortal tea” or “Divine Che”. Later, this beverage spread to other Far East countries, with bibliographic references from 414 AD reporting its export from Korea to Japan to treat Emperor Inkyo’s digestive problems [7,8]. From there, its consumption spread to regions of East Asia and Eastern Europe (19th and 20th centuries), acquiring regional adaptations and distinct names (e.g., “Manchurian Mushroom Tea”, “Tea Fungus”, “Kargasok Tea”, “Grib tea kvass”, “Indian Tea Fungus”, “Manchu Fungus”, “Teakwass”, “Tea Beer” and many others) [9]. Its production was traditionally artisanal, passed down through generations, which explains the regional variations in preparation methods observed around the world [6,10]. Industrial production and commercialization, however, are relatively recent, beginning in the 1990s, primarily in the United States, and leading to the establishment of organizations such as Kombucha Brewers International, which today comprises numerous commercial producers worldwide, including 134 kombucha-producing companies in the United States, 28 in Canada, and smaller numbers in Europe, where the largest producers are Spain and the United Kingdom [11]. The number of producers is expected to increase. In fact, between 2014 and 2018, the market for this beverage grew by approximately 20% annually, with a similar trend observed in subsequent years’ (USD 1.5 billion in 2018, USD 1.67 billion in 2019, USD 2.2 billion in 2020, and 3.5 to 5 billion in 2025) [4,11]. This increase is attributed to global consumption increasing demand of natural products with potential health benefits, particularly in the case of processed beverages and probiotic products. However, it is important to note that although some of these products are widely consumed as “functional foods” due to their potential health benefits, no health claims have been officially authorized by regulatory bodies such as the European Food Safety Authority. Moreover, there is a scarcity of clinical trials evaluating the effects of kombucha or its constituents on human health [12].

Additionally, the continued growth of the sector has led to modifications in the traditional composition of kombucha, resulting in alternative products often marketed under the same name. Many of these emergent alternatives use new raw materials, new microorganisms, or different fermentation conditions during beverage production [4,13]. Consequently, it is important to determine the key factors affecting the biological activities of this product, and how changes in individual components may affect the fermentation process itself, and consequently, the health benefits of the final, commercial products. This review article presents a comprehensive analysis of kombucha, addressing its chemical composition, with an emphasis on the bioactive compounds responsible for its potential health effects. The main biological properties reported in in vitro and in vivo studies are reviewed, as well as recent innovations in its production and emerging applications in food, biotechnology, and sustainable materials. Furthermore, aspects related to food safety and regulation are discussed, considering the challenges posed by the variability of the fermentation process and the need to establish clear criteria for its production and marketing.

## 2. Methodology

The literature included in this review was collected through searches in major scientific databases, including Web of Science, Scopus, and PubMed. The search was conducted using combinations of keywords such as “kombucha”, “fermented tea”, “SCOBY”, “bioactive compounds”, “fermentation”, “bioactivity”, “functional beverage”, “innovation”, “safety”, and “legislation”. Particular attention was given to peer-reviewed articles published during the last decade, although earlier studies were also included when they provided important background information. Both original research articles and review papers addressing the chemical composition, microbial ecology, fermentation parameters, biological properties, and technological aspects of kombucha production were examined. The final selection of references was based on their scientific relevance and contribution to the understanding of kombucha as a functional beverage.

## 3. Chemical Composition of Kombucha

The composition of this type of beverage varies considerably depending on the production method used—the choice of microorganisms and the type of tea employed being crucial factors. However, in general terms, kombucha is primarily composed of sugars, proteins, vitamins, minerals, phenolic compounds, organic acids (e.g., acetic, lactic, gluconic, glucuronic, and oxalic acids), and other minor compounds (e.g., alcohol) [7,12,14].

### 3.1. Nutritional Composition

**Figure 2:**
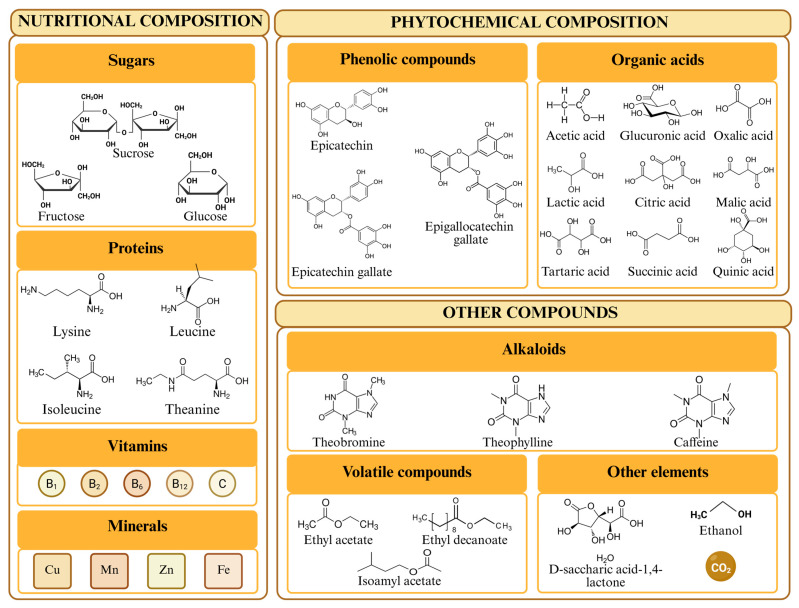
Chemical composition of kombucha.

Kombucha has a low energy content and a limited proportion of macronutrients (Figure 2), including residual sugars from fermentation [15]. During kombucha fermentation, most of the sucrose added in the initial steps is partially metabolized, resulting in varying concentrations of glucose and fructose [16], whose abundance depends on fermentation conditions and differs markedly between studies [5]. For example, one study found that the highest glucose levels were reported 14 days after the start of fermentation, with a concentration of up to 19.60%, while the maximum fructose concentration was 10.25% on the tenth day [17]. Similar results were obtained in another study in which fructose was metabolized before glucose, with all three sugars present after 21 days of fermentation [18].

In addition to sugars (Table 1), significant proportions of proteins have been identified, reaching contents of up to 3 mg/mL [5]. Regarding the composition of these proteins, it has been observed that most of the identified amino acids come from tea leaves, although some were also generated during fermentation by the metabolic activity of microbial SCOBYs in kombucha [19]. The main amino acids identified were lysine, leucine, isoleucine, and theanine, the latter representing up to 50% of the total amino acids [19,20]. At lower concentrations, it was possible to detect the amino acids alanine, aspartic acid, glutamic acid, methionine, phenylalanine, proline, threonine, tryptophan, and valine [20]. The presence of these compounds requires the study of the possible effects of microbial decarboxylation during fermentation, capable of transforming amino acids into different types and concentrations of biogenic amines [21]. Of the amino acids identified in kombucha, three can give rise to biogenic amines (lysine, phenylalanine, and tryptophan) [22]. Ethylamine, choline, and adenine are the only identifiable biogenic amines found in kombucha that have been discussed in the literature, none of which are harmful [23]. Nevertheless, information on this subject remains scarce, and the exact biotransformation route and the microorganisms involved are unknown [24].

In addition, this type of beverage is also a significant source of vitamins and minerals. For example, kombucha prepared from black tea has been shown to contain 74 mg/100 mL of vitamin B1, 52 mg/100 mL of vitamin B6, and 84 mg/100 mL of vitamin B12 [25], and 8.3 mg/100 mL of vitamin B2 [26]. Kombucha is also a significant source of additional B vitamins such as vitamins B3, B5, B7, B9 and vitamin C or ascorbic acid, with concentrations reaching 28.98 mg/L after 10 days of fermentation [23,26]. It should be noted, however, that vitamin levels reported in kombucha can vary considerably among studies due to differences in fermentation conditions, SCOBY composition, and analytical methods used for their determination. Similar variability has also been reported for other components of kombucha, including minerals and secondary metabolites such as organic acids and phenolic compounds. As for minerals, the tea leaves used play a fundamental role in the mineral composition of the beverage, as they constitute a significant source of iron, potassium, and manganese [27,28]. Other minerals identified in kombucha samples include cobalt, copper, magnesium, nickel, zinc, and fluoride ions [25,29], with concentrations ranging from 0.004 μg/mL to 0.462 μg/mL for cobalt and manganese, respectively [25]. Furthermore, the anionic characterization revealed that fluoride is the predominant anion, registering a concentration of 3.2 mg/g [30]. However, these fluoride levels will depend on the type of water used to make the beverage. For example, municipal water sources increase the concentration of this compound in the samples [27], the recommended dietary allowance for adults being 3–4 mg/mL for women and men, respectively [23]. Therefore, in some cases, a single glass of this beverage will constitute a significant contribution to this recommended amount. Furthermore, it has been shown that levels of copper, iron, manganese, nickel, and zinc can increase during the fermentation process, while cobalt levels decrease [23]. These studies also analyzed the presence of toxic minerals such as lead (0.005 μg/mL), chromium (0.001 μg/mL), and cadmium (below detection limit), all of which were considered safe levels for human consumption [25].

### 3.2. Phytochemical Composition

#### 3.2.1. Phenolic Compounds

From a functional perspective, phenolic compounds are among the main bioactive constituents of kombucha, originating from plant-based substrates—either tea or another herbal infusion (up to 30% of the dry weight in the tea leaves) [31,32]. Their levels are influenced by the SCOBY’s microbial composition, fermentation conditions (e.g., time, temperature, sucrose concentration), and the methods used for their analysis [20,23].

During fermentation, these compounds undergo transformations that may modify their chemical structure and bioavailability [33,34]. However, its metabolic pathway is not yet fully understood, although various lines of evidence suggest that SCOBY can hydrolyze complex phenolic compounds present in the plant ingredients used, releasing simpler forms that often increase the total content of soluble phenolic compounds. Furthermore, the presence of enzymes produced during fermentation—such as cellulases, glucosidases, and pectinases—can break down cell walls and polyphenolic complexes, facilitating their release into the liquid medium [35]. This would be the main reason behind the rise in total phenol concentration (up to 3.53 times) as fermentation progresses [36].

This increase in concentration is fastest during the first seven days of fermentation, which may be due to the higher microbial diversity achieved by that time [37,38], reaching maximum levels of epicatechin (~150%) and epicatechin gallate (~115%) in green and black tea on day 12 of fermentation [39]. The main contributors to this increase are phenolic compounds such as gallic acid, caffeic acid, epicatechin, and rutin, as well as phenolic compounds not present in the initial infusion such as chlorogenic, p-coumaric, and ferulic acids [36]. However, from day 21 of fermentation onwards, the concentration of these types of compounds begins to decrease significantly [40]. Such effects arise from other phenolic compounds, such as complex flavonoids, tend to decrease in concentration due to their transformation or microbial degradation [36]. This decrease in the concentration of phenolic compounds is also observed when red algae are used during the second fermentation [41]. However, further kinetic studies are still needed to fully understand the biotransformation processes of polyphenols during kombucha fermentation. To enhance this understanding, the development of suitable synthetic microbial communities would be of great interest. These communities would reveal the phenolic metabolic characteristics of complex microbial communities and allow for the creation of customizable phenolic combinations for kombucha products [42].

Regarding the phenolic profile of kombucha (Figure 2), kombucha made with black tea typically exhibits a greater overall diversity of phenolic compounds compared to that made with green tea (1.09 vs. 0.70 mg GAE/mL, respectively), resulting in a higher antioxidant capacity [43,44]. These values can be increased up to 5.68 times when filtration processes are not used during production [45]. Regarding the characterization of these phenolic compounds, more than 127 compounds have been identified in green and black tea kombucha, mostly flavonoids (70.2%), followed by phenolic acids (18.3%), other polyphenols (8.4%), lignans (2.3%) and stilbenes (0.8%) [43]. Among the flavonoids, catechins and their derivatives stand out (more abundant in green tea), including epicatechin, epicatechin gallate, and epigallocatechin gallate, with concentrations reaching 120–380 μg/L GAE [43,44,45,46]. Both epigallocatechin gallate and epicatechin gallate remain relatively stable during fermentation (18% and 23%, respectively). In contrast, catechin derivatives such as theaflavins and thearubigins are more susceptible to degradation, likely due to the activity of as-yet-uncharacterized enzymes released by yeasts and bacteria in the kombucha culture [39]. Other phenolic compounds identified include gallic acid, caffeine, gallocatechin gallate, gallocatechin, ellagic acid, quercitrin, astragalin, and isorhamnetin [45,47]. Kombucha prepared from black tea contains anthocyanins such as delphinidin-3-O-beta-d-galactoside, cyanidin-3-O-beta-d-galactoside, delphinidin-3-O-beta-D-[6-(E)-p-coumaroyl] galactopyranoside, and cyanidin-3-O-beta-D-[6-(E)-p-coumaroyl]; galactopyranoside are also detected [48]. When yerba mate is used as the substrate in the secondary fermentation, the resulting kombucha is enriched with additional phenolic compounds such as caffeoylquinic acid, dicaffeoylquinic acid, different flavonoids, and xanthines [40].

#### 3.2.2. Organic Acids

The main organic acids identified are acetic acid (0.21 to 16.57 g/L), glucuronic acid (0.04 to 7.36 g/L), lactic acid (0.015 to 0.702 g/L), citric acid (0.05 to 2.77 g/L), tartaric acid (0.23 g/L), succinic acid (3.05 g/L), oxalic acid (0.04 to 0.044 g/L), malic acid (0.029 to 0.03 g/L), and quinic acid (0.46 to 0.47 g/L); their concentration varied according to the type of tea and the fermentation conditions [24]. In most cases, the most abundant organic acid is acetic acid (16.57 g/L, reaching 3 g/L after 10 days of fermentation) followed by succinic acid (3.05 g/L), with the remaining identified compounds present in significantly lower concentrations [24,43]. However, when kombucha is made with green tea, the predominant organic acid is gluconic acid, with succinic, citric, and gallic acids being less abundant [48]. In fact, this is the type of kombucha formulation in which the highest concentrations of lactic acid are detected [23], being detectable only on the third day of fermentation [39]. Other organic acids identified in kombuchas made from tea include gluconic, folic, carbonic, malonic, pyruvic, and usnic acids [1,20,24]. Regarding the organic acids present in kombucha analogs, these mainly include acetic acid (up to 15.10 g/L), lactic acid (up to 5.25 g/L), glucuronic acid (up to 132.81 g/L), citric acid (up to 1072 g/L), malic acid (up to 1.59 g/L), oxalic acid (up to 3.45 g/L), formic acid (up to 2.33 g/L) and succinic acid (up to 0.30 g/L), with their concentration varying according to the type of substrate and the fermentation conditions [24]. For example, when grapes effluent is used, the predominant organic acid is acetic acid [49].

Concerning the kinetics of these compounds, acetic acid tends to slowly increase its concentration during fermentation. For example, when black tea is used, the concentration of this compound increases to 11 g/L after 10 days of fermentation, and then gradually decreases to 8 g/L after 60 days [50]; results were similar to previous studies (maximum 9.5 g/L after 15 days of fermentation) [39]. Another study observed an increase in acetic and gluconic acids concentration in green tea kombucha reaching 16.57 and 7.36 g/L, respectively, after 21 days of fermentation [37]. Glucuronic acid reaches its maximum in kombucha made with green tea after 12 days of fermentation (2.3 g/L) [39]. These trends were also observed when different herbal infusions were used for kombucha production. For example, formulations made with lemon balm showed a peak concentration of organic acids on the seventh day of fermentation, with a concentration of 8.12 g acetic acid/L [51]. In the case of soy whey, acetic and glucuronic acid concentration reached 5.77 and 132.81 g/L after 8 days of fermentation [52]. The subsequent gradual decrease in the concentration of organic acids is attributed to the fact that, once the available sugars in the formulation are depleted, the bacteria begin to use other carbon sources, including the organic acids themselves. In turn, the SCOBY’s metabolism causes the pH of the formulation to decrease, reducing the activity of the acetic acid bacteria [50]. In kombucha made with beet molasses, a greater decrease in pH and a higher total acid concentration (0.9–3.1 g/L) were observed compared to that prepared with sucrose, because molasses contains its own organic acids. However, acetic acid production was lower, possibly because some components of molasses inhibit acetic acid bacteria or promote the conversion of acetic acid into other compounds [53]. Therefore, it is necessary to optimize the fermentation time, since the accumulation of organic acids can exceed the limits considered safe for human consumption [5]. This optimization process must take into account factors such as temperature, since high values increase the fermentation rate and favor the accumulation of acids [41].

### 3.3. Others

#### 3.3.1. Alcohol

The main alcohol reported in kombucha is ethanol (Figure 2), which is generated during fermentation. Its concentration typically ranges from 0.007% to 2.3%, depending on the initial sugar content of the unfermented beverage, as well as storage conditions and the fermentation time and temperature [24,37,54,55]. Therefore, it is essential to control all these factors so that the resulting beverage does not exceed the maximum legal percentage of ethanol allowed in non-alcoholic beverages, with the average value taken as the limit being 0.5% (*v*/*v*) [24]. Otherwise, the beverage should be labeled as an alcoholic beverage, although it is increasingly common to find kombucha beverages with a higher alcohol content (known as “hard kombucha” and up to 5.5%) that are not labeled correctly [23,56]. This is of great importance when using black tea, as it undergoes a faster increase in ethanol concentration than green tea (0.3% versus 0.2% after 15 days of fermentation) [57]. Some studies show that this alcohol content can be reduced by increasing the fermentation time by one week, especially when acetic bacteria are used [37,58]. However, longer fermentation times (up to 60 days), regardless of the fermentation temperature, would not produce this decrease in alcohol content, with all samples having alcohol levels higher than those permitted in non-alcoholic beverages [55]. Therefore, to market these beverages without exceeding permitted alcohol levels, it is essential to properly control the fermentation process and store the product at appropriate temperatures, as refrigeration temperatures stabilize alcohol production by reducing the fermentation speed of viable microbial cells in the beverage [56].

#### 3.3.2. Alkaloids

Several studies have reported alkaloids in kombucha. These alkaloids originate from the tea leaves used as raw material, which are a significant source of caffeine (up to 3–6% of the content of tea leaves) [59]. These leaves also contain other alkaloids in lower concentrations (Figure 2), notably theobromine and theophylline [23,60]. All these alkaloids belong to the methylxanthine group, and their final concentration is influenced by the type of tea, the fermentation time, brewing conditions, and growing conditions of the raw material. They are characterized for being stable during the fermentation process [61]. During kombucha fermentation, caffeine contributes to the growth and metabolism of yeasts and bacteria by providing nitrogen for cell synthesis and the energy needed for the microorganisms to carry out fermentation [23].

#### 3.3.3. D-Saccharic Acid-1,4-Lactone

D-saccharic acid-1,4-lactone is an oxygenated organic compound that belongs to the lactone group, derived from the oxidation of carbohydrates during the fermentation process [62]. Its presence in kombucha is due to the metabolism of D-saccharic acid (glucaric acid) by the microorganisms that make up the SCOBY. It is considered a bioactive component of interest due to its chemical stability and its relationship with organic acid metabolism [63]. The presence and concentration of D-saccharic acid-1,4-lactone in the fermented beverage depend on factors such as the type of substrate, the fermentation time, and the process conditions [64], with contents ranging between 57.99 and 132.72 μg/mL, reaching the highest values on the eighth day of fermentation [65].

#### 3.3.4. Volatile Compounds

During kombucha fermentation, a series of volatile compounds are generated. These compounds are responsible for the characteristic sensory properties (especially fruity and floral notes) of this type of beverage. Among the various volatile compounds identified in kombucha, significant concentrations of complex mixtures of esters, alcohols, carboxylic acids, aldehydes, ketones, phenols, and terpenes—with esters such as ethyl acetate, ethyl decanoate, and isoamyl acetate—were detected. These compounds arise primarily from the metabolic activity of the SCOBY and vary depending on the raw materials, fermentation conditions, and storage time, which explains the aromatic differences between batches and fermented varieties [35,66].

## 4. Bioactive Properties of Kombucha

Kombucha has long been consumed as a beverage believed to promote health, and its regular intake has been linked to various potential benefits [24]. Its historical use as a traditional functional drink has prompted extensive research into its bioactive components and health-promoting properties. In the following subsections, the main bioactivities reported for kombucha will be examined, including its antioxidant, anticancer, and antimicrobial effects.

### 4.1. Antioxidant

Numerous studies, largely conducted in vitro, have demonstrated that different types of kombucha possess significant antioxidant activity. This activity is primarily attributed to the presence of various phenolic compounds and organic acids. However, most currently available studies have not isolated the potential bioactive compounds (at best, only the purification of the extracts is carried out) (Table 2). Therefore, this correlation between compounds and bioactivities can only be established statistically, without directly attributing the activity to individual molecules. Regarding the effectiveness of these extracts, kombucha fermentation has been reported to significantly improve the in vitro antioxidant capacity of the extracts due to the increased bioavailability of the bioactive molecules. Furthermore, during fermentation, some conjugated phenolic compounds undergo bioconversion, resulting in free phenolics and a consequent increase in antioxidant capacity [15]. Furthermore, the antioxidant potential is significantly influenced by factors such as the type of tea, fermentation time, and processing conditions, suggesting a joint contribution from polyphenols, organic acids, and other metabolites generated during fermentation [67,68]. For example, prolonged fermentation times (more than 14 days) could decrease the antioxidant capacity [69,70]. In the case of the sample formulation, it was observed that the antioxidant activity of the beverage was improved when brown sugar, white sugar, glucose and sucrose were used as sugars, obtaining significantly worse results with the use of artificial sweeteners [71]. Similar results were obtained with kombucha analogs. For example, kombucha tea fermented with coconut palm sugar exhibited higher in vitro antioxidant activity than with white refined sugar or molasses sugar [69]. Further examples can be found in Table 2, which shows that most of the available evidence comes from in vitro assays, limiting the direct extrapolation of these results to in vivo antioxidant effects in humans. However, according to the available studies, the antioxidant activity of kombucha appears to be mediated by multiple mechanisms. These include the ability to chelate pro-oxidant metals, which would otherwise catalyze oxidation reactions, and the modulation of pro-oxidant enzymes, which reduces the generation of reactive species. These actions act synergistically to neutralize free radicals, as observed in in vitro DPPH and ABTS assays [35].

### 4.2. Anticancer

The anticancer activity of kombucha has been extensively investigated, primarily through in vitro assays (Table 2). The observed effects have been associated with the combined action of phenolic compounds, organic acids, and other metabolites generated during fermentation (e.g., D-saccharic acid 1,4-lactone, dimethyl 2-(2-hydroxy-2-methoxypropylidene) malonate, vitexin) [63,68,79,90]. However, the composition of kombucha varies significantly between different preparations, which directly influences its bioactivity. In a study that evaluated human lung adenocarcinoma epithelial cells (A549), ileocecal colorectal adenocarcinoma cells (HCT8), colorectal adenocarcinoma epithelial cells (Caco-2), and normal lung cells (IMR90), kombucha made from green tea produced the most notable effects. This activity could be explained by its high catechin content and the presence of verbascoside—compounds associated with anticancer and antioxidant properties [43]. The anticancer properties of these extracts are attributed to their ability to inhibit cell proliferation, altering the cell cycle, and activating apoptotic pathways [63,68,79,90]. For example, kombucha treatment increased early apoptosis and induced cell cycle arrest at the G0/G1 phase in human colorectal cancer cells (HCT-116) more effectively than green tea extract alone. Mechanistically, this was accompanied by upregulation of tumor suppressor genes such as *p21* and *p53* and pro-apoptotic *Bax*, with concurrent downregulation of anti-apoptotic *Bcl-2*, suggesting that kombucha can influence gene expression linked to programmed cell death pathways when combined with chemotherapeutics [91]. In another study, kombucha treatment leads to increased apoptosis via modulation of *p53* and *Bax/Bcl-2* balance and results in cell cycle inhibition at G2/M phase in human liver cancer (HepG-2) cells, without obvious cytotoxicity against non-cancerous cells [102]. Moreover, some of the compounds present in kombucha have demonstrated in vitro the ability to modulate oxidative stress and the activity of enzymes involved in detoxification processes, which may indirectly contribute to antitumor effects [103,104]. In addition, recent research has focused on postbiotic metabolites produced by the kombucha microbiota. Specifically, cell-free supernatants obtained from strains of *Gluconobacter oxydans* isolated from kombucha demonstrated antiproliferative effects against gastric adenoma cells. These findings suggest that certain bioactive compounds produced by the microbial community during fermentation can exert cytotoxic activity independently of the complete kombucha drink [105]. However, the available evidence comes mostly from in vitro trials, so these results should be interpreted with caution and firm conclusions should not be drawn about anticancer effects in humans.

### 4.3. Antimicrobial

The antimicrobial activity of kombucha has been extensively documented in experimental studies, primarily through in vitro assays against pathogenic bacteria and spoilage microorganisms (Table 2), so its relevance under in vivo conditions still requires further investigation. This effect is attributed to the presence of organic acids, especially acetic acid, which lowers the pH of the medium and interferes with the integrity of the microbial cell membrane [12]. Furthermore, the presence of other bioactive compounds (e.g., polyphenols) or ethanol may exert synergistic effects [12]. The concentration of these compounds, and therefore the antimicrobial potential, will depend on the fermentation time, the type of tea, and the composition of the substrate, generally increasing as fermentation progresses [106]. For example, the addition of other vegetable ingredients such as cinnamon increases the concentration of some organic acids (e.g., acetic and glucuronic acids) with the consequent increase in the antimicrobial activity of kombucha [15,78,81]. Similar effects have been reported when plant matrices rich in phenolic compounds are added during secondary fermentation. For example, the addition of soy whey results in increased flavonoid content and antibacterial activity against *Staphylococcus aureus*, *Bacillus subtilis*, and *Escherichia coli* [52].

In addition to its direct antimicrobial activity, kombucha may exert functional effects through the modulation of the gut microbiota, an aspect of particular relevance in the field of functional foods [107]. Although evidence is still limited, recent studies suggest that kombucha consumption may induce changes in the host’s microbial composition. In a murine model of non-alcoholic fatty liver disease, modifications were observed in gut bacterial populations, with reductions in the abundance of genera such as *Allobaculum*, *Turicibacter*, and *Clostridium* after kombucha tea ingestion, changes that could contribute to decreased hepatic lipid accumulation [108,109]. These effects could be related to both the antimicrobial activity of certain kombucha metabolites and indirect interactions with the gut microbiota. However, extrapolating these results to humans requires caution, and controlled clinical trials are needed to conclusively assess the impact of kombucha consumption on the human microbiota and its implications for health.

### 4.4. Others

In addition to the bioactivities mentioned previously, various studies have demonstrated that kombucha possesses other bioactivities that contribute to human health, most of which are related to its antioxidant capacity. These effects include anti-inflammatory, hepatoprotective, and antidiabetic properties, as well as benefits in regulating metabolism and the immune system, reducing inflammation, and supporting overall physiological functions, thus reinforcing its appeal as a functional beverage [19].

Its anti-inflammatory effects (Table 2) are related to the reduction in pro-inflammatory mediators and the attenuation of oxidative stress (e.g., in vitro inhibition of 5-lipoxygenase activity or reduction in pro-inflammatory cytokines secretion), processes in which phenolic compounds and other metabolites generated during fermentation play a relevant role [68,79,85,110]. Therefore, kombucha could play a key role in modulating cellular pathways associated with the inflammatory response, with a potential protective effect against chronic inflammatory processes. However, despite these promising in vitro and in vivo results, evidence in humans remains limited [19].

Regarding the hepatoprotective activity of kombucha, several studies using animal models have reported improvements in biochemical parameters related to liver function and a reduction in histological alterations in the liver. In fact, kombucha has demonstrated a superior hepatoprotective effect to that of unfermented black tea in various animal models. For example, studies in albino rats exposed to aflatoxin B1 showed that kombucha reduced markers of hepatotoxicity such as aspartate transaminase, alanine transaminase, alkaline phosphatase, and gamma-glutamyl transpeptidase, decreased lipid peroxidation, and modulated the hepatic antioxidant system, including reduced glutathione and antioxidant enzymes such as glutathione S-transferase, glutathione peroxidase, glutathione reductase, catalase, and superoxide dismutase [111,112]. Furthermore, in murine models of non-alcoholic fatty liver disease and steatohepatitis, kombucha consumption protected hepatocytes against lipotoxicity, modulated lipid metabolism, and attenuated inflammation and fibrosis, contributing to liver restoration [113]. Part of this effect could also be related to the modulation of the gut microbiota, since kombucha tea has been observed to promote microbial populations that help reduce non-alcoholic fatty liver disease in mice [108]. These effects have been attributed to the combined action of antioxidants, organic acids, and other bioactive compounds capable of mitigating oxidative damage and promoting hepatic detoxification mechanisms. For example, glucuronic acid can bind to toxic substances in the liver, facilitating their efficient excretion [114]. Another compound of interest present in kombucha is D-saccharic acid 1,4-lactone, produced by *Gluconacetobacter* sp. during the fermentation process, which has detoxifying and hepatoprotective properties [23].

Furthermore, studies in animal models of induced diabetes suggest that kombucha may have antidiabetic effects (Table 2). In these studies, kombucha consumption has been associated with a reduction in blood glucose levels and an improvement in the lipid profile—effects that could be related to the modulation of glucose metabolism and insulin sensitivity [12,99]. For example, tea kombucha reduces the activity of plasma and pancreatic α-amylase [100]. This type of beverage is also capable of inhibiting porcine pancreatic α-amylase, increasing the inhibitory capacity with fermentation time and the presence of phenolic compounds (i.e., epigallocatechin gallate, gallocatechin gallate and epicatechin gallate) [57]. Another study observed that kombucha was rich in D-saccharic acid 1,4-lactone, which is a compound capable of protecting against hyperglycemia-induced hepatic apoptosis in diabetic rats [62]. However, the available evidence is still insufficient to draw firm conclusions in humans, with only one study of this type reported. In that study, kombucha was administered daily for three months to various consumers, and normalized blood sugar values were observed in subjects with non-insulin-dependent diabetes mellitus, but without considering a control group [12]. Therefore, controlled clinical trials are required.

Furthermore, several studies in animal models have indicated that kombucha may contribute to reducing total cholesterol, low-density lipoprotein cholesterol, and triglyceride levels, as well as increasing high-density lipoprotein cholesterol, suggesting a potential cholesterol-lowering effect (Table 2). This effect has been associated with the combined action of organic acids, polyphenols, and other bioactive metabolites produced during fermentation, which can modulate lipid metabolism and promote cholesterol excretion. Trials in hypercholesterolemia models have also shown improvements in liver parameters and a reduction in lipid accumulation in the liver, indicating a possible cardiovascular and metabolic benefit [100,101].

## 5. Innovations in Kombucha Formulation

The artisanal production of kombucha presents several challenges, most notably the lack of reproducibility between different production batches. This lack of uniformity leads to problems with stability and consistent bioactivity, hindering its commercialization and the declaration of functional properties. To overcome these challenges, several innovations in the production process have been implemented, including variations in fermentation conditions (time, temperature, concentrations, and microorganisms involved) and changes in substrate composition (use of different starting matrices or fortification). All these parameters directly affect microbial dynamics and, therefore, the accumulation of metabolites and the composition of the final product [115,116,117].

Regarding the parameters that influence fermentation, the most important are temperature, pH, oxygen, substrate, sugar concentration, SCOBY origin, container geometry, and fermentation time. Any variation in these factors can interfere with sensory characteristics, chemical properties, nutritional quality, and biological activities [5]. In most cases (Table 3), the sugar used is sucrose at a concentration of 20%. The concentration of tea or herbal infusion usually varies between 1.0 and 100 g/L [24]. Regarding SCOBYs, this is one of the main areas of innovation in the kombucha industry. The combinations of microorganisms that can make up a SCOBY are endless due to the inherently diverse microbial composition of kombucha (Table 4). In most cases, the most abundant microorganisms are bacteria belonging to the genus *Komagataeibacter* and yeasts from the genera *Zygosaccharomyces*, *Brettanomyces*, or *Saccharomyces* [19]. This knowledge has served as the basis for exploring custom-designed consortia that combine microorganisms with complementary metabolic functions. This is the case with SCOBYs composed of cellulose-producing bacteria and ethanol-resistant probiotic yeasts [118,119]. In this respect, the latest available studies show that isolating and combining specific microbial strains allows for increased production of certain desirable bioactive metabolites, as well as more consistent results after each fermentation [120]. For example, the combination of *Komagataeibacter swingsii*, *Komagataeibacter saccharivorans* and *Dekkera bruxellensis* allows for up to three times the levels of gluconic acid in the resulting beverage, with the consequent reduction in pH and an increase in the functional potential of the final product [121,122]. Another study reported that selecting strains such as *Komagataeibacter saccharivorans*, *Brettanomyces anomala*, and *Kluyveromyces marxianus* resulted in non-alcoholic kombucha without diminishing its probiotic capacity or sensory quality during over 60 days of storage [123]. It has also been observed that including probiotics such as *Lactiplantibacillus plantarum* or *Pediococcus pentosaceus* can provide more robust probiotic functions, with the potential to improve gut health when the strains survive gastrointestinal transit [124]. Therefore, SCOBY optimization represents one of the main steps to achieve a reproducible fermentation process with the desirable functional properties. This optimization could also help in the prevention of the production of unwanted compounds (e.g., biogenic amines or high alcohol content) during fermentation, molecules that could compromise product safety and consumer acceptance [125]. This is especially relevant at the end of fermentation, when most of these unwanted compounds are produced. Therefore, thorough control of the fermentation process would not only reduce the potential risks of this beverage but also improve its possible functional attributes.

The selection of substrates is another fundamental aspect of the fermentation process, both in primary fermentation (carried out using tea) and in secondary fermentation, which continues after the removal of the SCOBY layer, as active microorganisms remain in the liquid. The addition of new matrices (Table 3) during secondary fermentation has proven to be an effective strategy for enriching the nutritional and functional profile of kombucha [24,141]. For example, kombuchas formulated with papaya and whey proteins encapsulated with probiotics showed improved microbiological stability, together with a greater capacity to inhibit α-glucosidase and enzymes related to lipid metabolism, suggesting that the addition of nutrient matrices can broaden the spectrum of functional activities beyond the traditional properties of kombucha [52,134]. The applicability and potential of these alternative formulations stem from the fact that the microorganisms in the SCOBY not only consume the available carbon in the medium but also metabolize many of the other compounds present in the mixture. This metabolism encompasses diverse metabolic pathways that give rise to a wide variety of bioactive compounds. Therefore, the addition of other raw materials not only helps diversify the kombucha market and attract consumers with new sensory properties but would also allow for the development of products with optimized functional properties. This would include beverages with anti-inflammatory potential or metabolic support. For example, infusion of lemon balm (*Melissa officinalis* L.), pear juice (*Opuntia ficus-indica*), or yarrow (*Achillea millefolium*) fermented with kombucha culture showed higher antimicrobial and antioxidant activity compared to the unfermented infusion [51,92,129,131].

**Table 4:** Microorganism species mainly found in SCOBY for kombucha fermentation.

Yeast	Bacteria	Fermentation	Ref.
*Zygosaccharomyces bailii*, *Schizosaccharomyces pombe*, *Torulospora delbreuckii*, *Rhodotorula mucilaginosa*, *Brettanomyces bruxellensis*, *Candida stellata*	*Acetobacter* spp.	Black tea//5 min, 100 °C, 1 L W, 100 g sucrose, 5.4 g/L tea//14 days, RT	[142]
*Zygosaccharomyces* sp., *Saccharomyces cerevisiae*	*Acetobacter* spp.	Green and black tea//5 min, 100 °C, 1 L W, 70 g sucrose, 1.5 g/L tea//10 days, 28 °C	[26]
*Dekkera bruxellensis*, *Dekkera anomala*, *Zygosaccharomyces bisporus*, *Zygosaccharomyces lentus*, *Pichia kudriavzevii*, *Davidiella tassiana*, *Wallemia sebi*, *Lachancea fermentati*, *Leucosporidiella fragaria*, *Kazachstania unispora*, *Kluyveromyces marxianus*, *Naumovozyma castelli*, *Hanseniaspora vineae*	*Lactobacillus*, *Lactococcus*, *Leuconostoc*, *Bifidobacterium*, *Acetobacter*, *Gluconacetobacter*	Black tea//15 min, 100 °C, 1 L W, 100 g sucrose, 0.49% *w/v* tea//10 days, 23 °C	[143]
*Candida*, *Lachancea*, *Kluyveromyces*, *Debaryomyces*, *Pichia*, *Waitea*, *Eromothecium*, *Meyerozyma*, *Zygowilliopsis*, *Saccharomyces*, *Saccharomycopsis*, *Hanseniaspora*, *Kazachstania*, *Starmera*, *Merimbla*, *Sporopachydermia*, *Sugiyamaella*	*Lactobacillus*, *Weissella*, *Bifidobacterium*, *Komagataeibacter*, *Gluconobacter*	Black tea//5 min, 100 °C, 1 L W, 100 g sucrose, 5 g/L tea//21 days, 28 °C	[37]
*Candida boidinii*, *D. anomala*, *D. bruxellensis*, *Hanseniaspora valbyensis*, *Wickerhamomyces anomalus*, *Pichia membranifaciens*, *S. cerevisiae*, *Saccharomyces uvarum*, *Torulaspora microellipsoides*, *Z. bailii*, *Zygosaccharomyces florentina*	*Oenococcus oeni*, *Lactobacillus nagelii*, *Lactobacillus satsumensis*, *Acetobacter lovaniensis*, *Acetobacter okinawensis*, *Acetobacter peroxydans*, *Acetobacter syzgii*, *Acetobacter tropicalis*, *Gluconacetobacter eurapaeus*, *Gluconacetobacter hansenii*, *Gluconacetobacter intermedius*, *Gluconacetobacter liquefaciens*, *Gluconacetobacter xylinus*, *Gluconobacter cerinus*, *Gluconobacter oxydans*, *Tanticharoemia sakaeratensis*	Black and green tea//CP//8 days, RT	[144]
*Dekkera*, *Pichia*, *Zygosaccharomyces*	*Lactobacillus*, *Leuconostoc*, *Lactococcus*, *Bifidobacterium*, *Gluconacetobacter*, *Acetobacter*	Black tea//5 min, 100 °C, 1 L W, 100 g artificial sweeteners, 0.1 g/L tea//7 days, 24 °C	[71]
Nd	*Lactobacillus*, *Lactococcus*, *Streptococcus*, *Acetobacter*, *G. xylinus*, *Gluconacetobacter saccharivorans*	Green tea//10 min, 100 °C, 1 L W, 100 g sucrose, 10 g/L tea//21 days, 20 °C	[145]
*B. bruxellensis*, *Zygosaccharomyces parabailii*	*Lactobacillaceae*, *Leuconostocaceae*, *Streptococcaceae*, *Komagataeibacter intermedius*, *Komagataeibacter rhaeticus*, *Gluconobacter entanii*	Green and black tea, roiboos//3 min, 74 °C, 1 L W, 80 g sucrose, 8 g/L tea//14 days, 27 °C	[86]
*Zygosaccharomyces*, *Z. bailii*	*Komagataeibacter*, *K. rhaeticus*	Nd//10 min, 100 °C, 1.35 L W, 150 g sucrose, 6.7 g/L tea//15 days, 28 °C	[146]
*Candida arabinofermentans*, *B. bruxellensis*, *S. pombe*, *Z. bailii*	*Acetobacter maolroum*, *Acetobacter pasteurianus*, *Acetobacter pomorum*, *Acetobacter tropicalis*, *K. rhaeticus*, *Komagataeibacter xylinus*, *Komagataeibacter europaeus*, *K. intermedius*, *G. oxydans*, *Gluconacetobacter* sp. SXCC-1	Black tea//15 min, 80 °C, 1 L W, 70 g sugar, 10 g/L tea//14 days, 25 °C	[68]
*Z. bailii*	*A. pasteurianus*, *G. xylinus*	Black tea//15 min, 100 °C, 1 L W, 100 g sucrose, 8 g/L tea//10 days, 29 °C	[147]
*B. bruxellensis*, *Zygosaccharomyces bisporus*	*Acetobacteraceae*, *K. rhaeticus*, *K. hansenii*, *K. xylinus*	Green tea//15 min, 121 °C, 1 L W, 70 g sucrose, 0.5% tea//15 days, 30 °C	[148]
*D. bruxellensis*, *S. uvarum*, *Z. bailii*	*L. nagelii*, *O. oeni*, *A. tropicalis*, *A. okinawensis*, *K. hansenii*, *G. oxydans*	Green tea//30 min, 104 °C, 1 L W, 55 g blond sugar, 0.5% tea//27 days, 25 °C	[149]
*B. bruxellensis*, *H. valbyensis*, *S. cerevisiae*	*Acetobacter papayae*, *Gluconobacter takamatsuzukensis*	Black tea//1 L W, 60 g sucrose, 1% tea//3 days, 26 °C	[150]
*B. bruxellensis*, *D. bruxellensis*	*G. intermedius*	Black tea//121 °C, 1 L W, 100 g sucrose, 1 g/L tea//8 days, RT	[151]
*Zygosaccharomyces* sp.	*Acetobacter xylinum*	Tea bag//10 min, RT, 1 L W, 70 g sucrose, 1.5 g/L tea//60 days, RT	[152]
Nd	*Acetobacteraceae*, *Gluconacetobacter*	CE	[153]
Nd	*Acetobacteraceae*, *Komagataeibacter*	CE	[154]

Abbreviatures. Nd: not determined; CE: commercial extract; RT: room temperature; W: water; CP: commercial preparation.

Furthermore, the design of fortified kombucha products benefits from a deeper understanding of how microbial and chemical interactions influence the stability of added components. For example, certain phenolic compounds can be preserved or even transformed into more active forms during fermentation, while others may degrade if conditions are not optimal [155]. Therefore, fortification must consider not only the timing of the addition but also the compatibility of the added compounds with the microflora and the overall fermentation process. Additionally, sensory evaluations should be conducted to assess the effect of fortification on consumer acceptance of the final product [156,157]. In some cases, the addition of these matrices has increased acceptance. For example, a kombucha SCOBY-fermented beverage made from grape juice showed better sensory acceptance and functional properties compared to the unfermented grape beverage after 6 days of fermentation [137]. Consequently, many companies are investing in new formulations with a wide range of flavors that combine fruits, spices, herbs, and botanical extracts, adapting to local tastes and global trends as a strategy to attract new consumers and retain existing ones. Furthermore, efforts have focused on improving texture and carbonation levels, key aspects for product acceptance, especially among consumers accustomed to traditional soft drinks [158].

Another key aspect is sustainability, which has become a central driver of innovation in this industry. Many companies are adopting more sustainable production practices, such as using organic ingredients, reducing water and energy consumption, and valorizing byproducts from the fermentation process [159]. SCOBY, for example, has been reused in the production of alternative products such as dehydrated snacks, supplements, or even biodegradable materials, reflecting an innovative approach to the circular economy [160,161].

Therefore, the combination of these strategies (i.e., fermentation optimization, including the selection of more efficient strains, the incorporation of alternative matrices, and/or fortification) is transforming kombucha formulation from a traditional art into a modern food biotechnology focused on reproducibility, functionality, and safety. These innovations are supported by a growing body of scientific literature that utilizes meticulous microbiological analyses, metabolomics, bioactivity studies, and experimental design approaches to guide formulation improvements. As more data emerges on how the microbiota interacts with ingredients and fermentation conditions, it will become possible to design fermented beverages with predefined functional profiles and specific health benefits, potentially further solidifying kombucha’s position within the functional food sector.

## 6. Safety and Regulations

Kombucha is a fermented beverage that is generally safe for human consumption when produced, stored, and consumed under hygienic and controlled conditions. However, increased production and consumption have raised concerns about its safety, both in industrial and artisanal settings. According to the International Association for Food Protection, the potential risks of kombucha can be grouped into chemical and biological hazards [19].

Regarding chemical hazards, one of the most frequently discussed risk is the ethanol content of kombucha. Although commercial products are usually labeled as non-alcoholic, alcohol is produced as a natural part of the fermentation process, including secondary fermentation in the bottle, which is rarely analyzed in studies. The concentration of ethanol depends on factors such as fermentation time, microbial composition, and storage conditions. In some cases, ethanol levels have been reported to exceed legal limits set for non-alcoholic beverages, with implications for consumer safety and regulatory compliance [1,23,56]. The permitted percentage of ethanol varies slightly from country to country. In the United States and Europe, a maximum of 0.5% is allowed at any stage of production, bottling, or storage [162]. A higher content, even on a one-off basis, would legally require that production batch to be classified as an alcoholic beverage, subjecting it to different regulations that include distinct production, licensing, labeling, and tax requirements specific to alcoholic beverages. To avoid this problem, many studies apply a pasteurization stage that eliminates any remaining microorganisms in the beverage [58]. Furthermore, the controlled addition of preservatives after fermentation could help stabilize the beverage by maintaining its characteristic pH and inhibiting the activity of residual yeasts [31,163]. This prevents further fermentation of remaining sugars during storage, which could otherwise lead to an increase in ethanol content and changes in acidity and flavor.

Another chemical concern relates to the formation of biogenic amines, such as ethylamine, choline, tyramin, Spermine, N-(9-oxodecyl) acetamide, N-(4-morpholinophenyl)-4-(1H-pyrazol-1-yl) benzamide, and adenine. These compounds can arise during the fermentation of proteins and amino acids by certain microorganisms [22,164]. At high concentrations, biogenic amines have been associated with adverse health effects, particularly in sensitive individuals or when consumed in large amounts [165]. Reported levels of amines in kombucha vary widely across studies, depending on factors such as the fermentation substrate and the microbial community. Concentrations typically increase from day 0 to day 6, then drop sharply on day 8, rise again on day 10, and decrease once more by day 12, which may correlate with the predominant presence of microorganisms such as *Lactobacillus parafarraginis*, *Lactobacillus harbinensis*, and *Lentilactobacillus*, which are the predominant microorganisms that grew up to day 6 [164]. Conversely, the decline in amine levels after day 6 may be attributed to the metabolic activity of other microorganisms, including *Saccharomyces* sp. and *Lactobacillus curvatus*, which become more abundant during the mid-stage of fermentation [164,166,167]. Therefore, understanding the microbial ecology and the metabolic pathways involved in the synthesis and degradation of biogenic amines is crucial for controlling their levels in kombucha. Moreover, given the potential presence of harmful biogenic amines during fermentation, future studies should investigate the relationship between specific microbial strains and the formation of these compounds, using functional experiments in which sterilized kombucha is inoculated with individual strains to quantify their amine production.

Therefore, these chemical hazards arise from the metabolic processes of the fermenting microorganisms, which underscores the need to control microbial growth and activity to prevent the occurrence of biological hazards. Their growth can occur at any stage of production emphasizing the need to carefully manage microbial activity [19]. Therefore, strict hygiene measures must be established to prevent the occurrence of microbial hazards, including the sanitization of fermentation vessels and tools, the use of previously boiled water for tea preparation, the selection of a high-quality uncontaminated SCOBY, pH monitoring, equipment cleaning, and the use of certified raw materials [19,168]. These practices help minimize the risk of undesirable microorganism proliferation by ensuring adequate hygienic conditions and pH control that limits their growth and significantly reduce the risk of growth of foodborne pathogens such as *Salmonella* spp., *Listeria monocytogenes*, *Bacillus* spp., *Staphylococcus aureus*, and *Clostridium botulinum*, or contamination by molds such as *Penicillium* and *Aspergillus* [169,170]. Nevertheless, in the case of kombucha, this risk is inherently low due to its acidic pH (generally between 2.5 and 4.2), which constitutes an intrinsic barrier that inhibits the growth of most foodborne pathogens. In fact, the Food and Drug Administration (FDA) in its Food Code Model establishes pH values of 4.2 as the maximum values on the seventh day of fermentation for kombucha to be considered microbiologically safe. Conversely, values below 2.5 necessitate restoring the beverage to an acceptable pH range [24,58] to avoid physiological damage and corrosion of vessels used to ferment and store them [19]. This erosion can generate the release of compounds with potential negative health effects (e.g., heavy metals) [171].

Therefore, both chemical and biological hazards in kombucha can be effectively minimized through careful control and standardization of the fermentation process. The degree of risk reduction largely depends on the production system, which highlights important differences between home-brewed and industrially produced kombucha. In home-brewed or artisanal preparations, there is often a lack of standardized procedures and comprehensive monitoring, being subject to greater variability in ingredients, fermentation conditions, and hygiene practices, which can increase the likelihood of safety issues if careful procedures are not followed [157,172]. Industrial production benefits from controlled fermentation conditions, systematic quality management, and adherence to regulatory standards, including the implementation of Hazard Analysis and Critical Control Points (HACCP) systems [168]. Such systems involve identifying potential hazards, defining critical control points in the production process, establishing monitoring procedures, and setting corrective actions to prevent or eliminate risks (including poorly controlled herbal ingredients or additives, contamination with resistant spores, generation of unwanted compounds, or high ethanol content), ensuring both product safety and consistency [173].

In addition to microbiological and chemical risks, isolated adverse effects associated with kombucha consumption have been reported. These occur primarily when the beverage is ingested in large quantities, prepared using poor hygiene practices, or consumed by vulnerable populations, such as young children, pregnant women, people with compromised immune systems (e.g., HIV infection, acute renal failure, or a predisposition to acidosis), and individuals with hypersensitivity or allergies to certain kombucha compounds. Furthermore, consuming highly acidic kombuchas, resulting from prolonged fermentation processes, may increase the likelihood of adverse effects [20,31]. Reported adverse effects include gastrointestinal problems, allergic reactions, mild liver toxicity, lactic acidosis, acid accumulation in the blood, hyponatremia, anti-Jo1 myositis, cholestatic hepatitis, jaundice, headache, and dizziness [19,23,31]. High consumption of kombucha can also negatively affect dental health due to its tannin content, which can accumulate on the teeth and lead to discoloration [174], as well as its acidity, which erodes tooth enamel, potentially leading to halitosis and enamel weakening, thus increasing the predisposition to tooth decay [175]. Furthermore, the added sugars used to promote the fermentation process can have an adverse effect on oral health [23]. These high sugar contents can also contribute to other health problems, such as obesity, cardiovascular disease, and diabetes [23]. In pregnant women, attention should be paid to the concentrations of glycosaminoglycans and heparin—compounds present in teas that are capable of inhibiting the blood-clotting system protein in the body. This is a critical aspect especially in the third trimester [20]. For these reasons, the bioactive compounds present in kombucha should be systematically quantified, and evidence-based guidelines for daily consumption should be established and communicated to consumers through product labeling [31,163]. Faced with these challenges, some producers have sought certifications for internationally recognized food safety standards, such as IFS Food in Europe or ISO 22000 management systems, which involve external audits, traceability, and systematic hazard control [176,177]. These certifications help ensure that the kombucha produced meets hygiene standards, raw material controls, documented processes, and labeling requirements that support product safety.

In this regard, a recent scientific review on the risks and toxicity of kombucha highlights that while most commercial kombuchas are safe, the lack of clear and uniform regulatory frameworks in many countries hinders the standardization of safe production practices [157,178]. Therefore, it is essential to develop appropriate regulations and labeling, incorporating food safety principles even in home settings, such as strict cleaning measures, the use of safe raw materials, pH measurement, and, where possible, validation of fermentation techniques through microbiological analysis. Currently, Brazil is the only country with specific legislation for kombucha, established through Normative Instruction No. 41/2019 of the Ministry of Agriculture, Livestock and Supply (MAPA). This regulation defines the official criteria regarding product name, composition, classification, labeling requirements, marketing restrictions, and analytical parameters applicable to this fermented beverage. These parameters include pH (2.5–4.2), alcohol content for non-alcoholic kombucha (≤0.5%) and alcoholic kombucha (0.6–0.8%), volatile acidity (30–130 mEq/L), and pressure in carbonated products. Furthermore, the regulation prohibits the use of labeling terms that suggest functional properties or superlative qualities not supported by current legislation [24]. In the United States, fermented drinks and acidified canned foods are exempt from regulation by the FDA; therefore, there is no specific legislation for kombucha, only recommendations established under the framework of the FDA Food Code, which establishes basic HACCP protocols applicable to the entire production chain [179]. For its part, Kombucha Brewers International has established a code of good practices that promotes food safety, quality and transparency standards for producers, in addition to a voluntary certification program [24]. In any case, the marketing of this beverage must comply with legal labeling requirements, including ingredients, net content, storage conditions and alcohol warnings if applicable.

## 7. Conclusions and Prospects

Kombucha is a fermented beverage whose final composition results from the interaction between the plant-based substrate and the metabolic activity of a complex microbial community. Throughout the fermentation process, various compounds are generated and transformed, including polyphenols and organic acids, which have shown biological activity in experimental studies, such as antioxidant or antimicrobial effects. However, the presence and concentration of these compounds vary considerably depending on the brewing conditions, which limits the generalizability of their potential effects and highlights the need for caution in interpreting the benefits attributed to this beverage. Current research suggests that the microbial consortium of kombucha and its cellulosic biofilm also hold potential for sustainable applications in packaging, environmental, and biomedical fields. Despite their renewable and low-cost nature, industrial-scale use requires careful evaluation of safety, functionality, and stability. Overall, both kombucha and its associated microbiota represent an expanding field of study that combines nutritional, technological, and biomaterial interests, but whose responsible use depends on standardized processes and more robust scientific validation.

## Figures and Tables

**Table 1:** Nutritional composition reported in kombucha.

Compounds	Fermentation Conditions	Concentration	Results	Ref.
**Sugars**				
Sucrose, glucose, fructose	Black tea//15 min, 80 °C, W, 70 g sucrose//21 days	Up to 19.60% glucose and 10.25% fructose.	Highest glucose and fructose levels at 14 and 10 days.	[17]
Inulin and its polyfructan fractions, sucrose, glucose, fructose	Jerusalem Artichoke tubers + black tea//15 min, 80 °C, W, 70 g sucrose//21 days	Up to 0.59% glucose and 10.41% fructose.	Much less content of glucose and fructose than kombucha without artichoke.	[17]
Sucrose, glucose, fructose	Black tea//5 min, 100 °C, W, 100 g sucrose//21 days	Up to 0.22 mol/L glucose and 0.10 mol/L fructose.	Maximum concentrations at 14th day of fermentation.	[18]
Sucrose, glucose, fructose	Black tea//5 min, 100 °C, W, 30 g sucrose//7 days	Up to 17.81 g/L sucrose, 9.35 g/L glucose, and 1.41 g/L fructose.	Fructose is preferred as the source of carbon by yeast.	[15]
**Proteins**				
Total proteins	Black tea//5 min, 100 °C, W, 100 g sucrose//15 days	Up to 3 mg/mL.	Originate from tea leaves.	[5]
Amino acids: lysine, leucine, isoleucine, theanine	Black tea//5 min, 100 °C, W, 100 g sucrose//21 days	Theanine up to 50% of total amino acids.	Minor quantities can be produced during fermentation.	[19,20]
Amino acids	Black tea//5 min, 100 °C, W, 100 g sucrose//21 days	Theanine up to 50% of total amino acids.	Trace levels of alanine, aspartic acid, glutamic acid, methionine, phenylalanine, proline, threonine, tryptophan, and valine.	[19,20]
Biogenic amines (ethylamine, choline, and adenine)	CE	NS	None are harmful.	[23]
**Vitamins**				
Vitamins B	Black tea//5 min, 100 °C, W, 70 g sucrose//15 days	B_1_: 74 mg/100 mL, B_6_: 52 mg/100 mL, B_12_: 84 mg/100 mL	Relevant source of vitamins.	[25,26]
Vitamin C	Black tea//5 min, 100 °C, W, 70 g sucrose//15 days	1.51 mg/mL	Relevant source of vitamins.	[25]
Vitamins B3, B5, B7, B9 and C	Black tea//5 min, 100 °C, W, 70 g sucrose//10 days	Up to 28.98 mg/L	All are water-soluble.	[23,26]
**Minerals**				
Iron, potassium, and manganese	Black and green tea//10 min, 90 °C, W, 100 g sucrose//14 days	Mn ≈ 1.57 mg/L; Fe ≈ 0.31 mg/L	Mineral profile largely reflects the composition of the tea substrate.	[27,28]
Cobalt, copper, magnesium, nickel, zinc, and fluoride ions	Black tea//5 min, 100 °C, W, 70 g sucrose//15 days	0.004 μg/mL to 0.462 μg/mL for cobalt and manganese, respectively.		[25,29]

Abbreviatures. CE: commercial extract; NS: not specified.

**Table 2:** Main bioactive properties reported in kombucha.

	Bioactive Compounds	Infusion	Model and Results	Ref.
**Antioxidant activity**				
In vitro (RSA)	PP//TP	Black tea//5 min, 100 °C, W, 100 g sucrose	Time dependent; ↑ up to 70%	[38]
	PP//TP	Black tea//5 min, 100 °C, W	↓ activity after 8 weeks	[72]
	PP//TP	Black tea//5 min, 100 °C, 1 L W, 20 g sucrose, garlic	IC_50_ 0.086 mL	[73]
	PP//TP	Black tea//10 min, 100 °C, 1 L W, 70 g sucrose	Tea (89.69%) > barley (76.19%) > rice (36.04%)	[67]
	PP//TP	Black tea//5 min, 100 °C, 1 L W, 100 g artificial sweeteners	↑ activity with brown sugar, white sugar, glucose, and sucrose	[71]
	PP//TP	Green, black tea//5 min, 100 °C, 1 L W, 100 g sucrose	50–80 µg GAE/mL	[74]
	PP//TP	Green tea//5 min, 100 °C, 700 mL W, 35 g sucrose, *Malvaviscus arboreus*	EC_50_ 80.7 mL/L	[75]
	PP//TP	Green tea//15 min, 100 °C, 1 L W, 70 g sucrose, bee pollen	IC_50_ 10.56–15.16 mg/mL	[76]
	PP//TP	Commercial preparation	No changes during shelf-life	[77]
	PP//HPLC	Green tea//15 min, 100 °C, 1 L W, 65 g sucrose, cinnamon, cardamom, or Shirazi thyme	↑ activity kombucha with cinnamon	[78]
	PP//HPLC	Black tea//5 min, 100 °C, 1 L W, 100 g sucrose, soy whey	EC_50_ 1.66–9.11 mg extract/mL	[52]
	PP//GC-MS	Black tea//HAE (15 min, 80 °C, W)	Activity not affected by the vessel geometry or the fermented volume. IC50 9–28.0 μg/mL	[79]
	PP//UHPLC-HRMS	Green tea//20 min, 100 °C, 1 L W, 100 g sucrose	Food preservative	[80]
	PP//UHPLC-QTOF-MS	Green and black tea//2–4 min, 75–95 °C, 1 L W, 50 g sucrose	↑ activity black tea	[43]
	PP, OA//TP, FTIR	Black tea//5 min, 100 °C, 1 L W, 30 g sugar	180.17 mg GAE/L	[15]
	PP, OA//CG-MS	Black tea//15 min, 80 °C, 1 L W, 70 g sugar	Non-polar phenolic compounds exhibited a higher activity	[68]
	PP, OA//HPLC, TP	Green, oolong, and black tea//15 min, 100 °C, 1 L W, 100 g sucrose	1.248–2.642 mg GAE/mL	[81]
	PP, OA, vitamin C, sugars//HPLC	Black tea//10 days, 28 °C, 1 L soymilk, 70 g sucrose	0.1 mL, 50%	[82]
	PP, OA, vitamin C, sugars//HPLC	Black tea//7 days, 25 °C, 1 L winery effluent, 70 g sugar	89%	[83]
	OA, vitamin C//HPLC	Green and black tea//5 min, 100 °C, 1 L W, 70 g sucrose	47.7%	[26]
In vitro (ACE)	Vitamin C//HPLC	Black tea//7 days, 25 °C, 1 L milk, 70 g sucrose	↑ ACE inhibition on 14th day of storage	[84]
In vitro (macrophages)	PP//LC-ESI-MS/MS	Black tea//10 min, 80 °C, 1 L W, 100 g sucrose, oak	↓ oxidative stress (2–200 μg/mL)	[85]
In vitro (RSA, cell cultures)	PP, OA//HPLC	Green and black tea, rooibos//3 min, 74 °C, 1 L W, 80 g sucrose	↑ activity in black and green kombucha; rooibos important effect on the recovery of oxidative damage on fibroblast cell lines against oxidative stress	[86]
In vivo (mice)	PP//TP	Black tea//5 min, 100 °C, W	Suppressed the oxidative stress mediated organ dysfunction in ALX induced diabetes	[87]
In vivo (mice)	PP//HPLC	Black tea//15 min, 100 °C, 1 L W, 100 g sucrose	Potent antioxidants to radicals and inhibitors of LDL oxidation	[88]
In vivo (rats)	Nd	Commercial preparation	↑ plasma and tissue MDA levels; ↓ DTH response, enhanced glutathione peroxidase, and catalase activities	[89]
**Anticancer activity**				
In vitro	PP//TP	Green tea//15 min, 100 °C, 1 L W, 70 g sucrose, bee pollen	Hep-2//IC_65_ 20 mg/mL	[76]
	PP//GC-MS	Black tea//15 min, 80 °C, W	MCF7, HCT-116//IC_30_ 50 μg/mL, IC_50_ 50 μg/mL in HCT-116	[79]
	PP//NMR	Black tea//5 min, 100 °C, 1 L W, 100 g sucrose	A549, U2OS, 786-O//0.25 μg/mL	[90]
	PP//UPLC-QTOF-MS	Green and black tea//2–4 min, 75–95 °C, 1 L W, 50 g sucrose	CACO-2, A549//low cytotoxicity and antiproliferative action against non-cancer cells; GI_50_ 40.93–47.15 and 116.8–137.0 μg/mL	[43]
	PP, OA//HPLC, TP	Green, oolong, and black tea//15 min, 100 °C, 1 L W, 100 g sucrose	CACO-2//IC_50_ 1.18–16.34%	[81]
	PP, OA//CG-MS	Black tea//15 min, 80 °C, 1 L W, 70 g sugar	HCT-116, MCF-7, OVCAR//50 μg/mL inhibition of up to 8.0, 14.6, and 34.7%	[68]
	Nd	Green tea//15 min, 100 °C, 1 L W	HCT-116//apoptosis induction and G0/G1 phase arrest, IC_50_ 0.9 mg/mL	[91]
	Nd	Tea leaves//15 min, 100 °C, 1 L W with lemon balm, 70 g sucrose	HeLa, MCF7, HT-29//IC_50_ > 500μg/mL	[92]
	Nd	Black tea//100 °C, 1 L W, 100 g sucrose	PC-3//IC_50_ 400 μg/mL	[93]
In vivo	Nd	Commercial preparation	Colon cancer//500 mg/kg	[94]
**Antimicrobial activity**				
In vitro (DDM)	PP//TP	Black tea//5 min, 100 °C, 1 L W, 20 g sucrose, garlic	*Staphylococcus saprophyticus*, *Staphylococcus aureus*, *Staphylococcus epidermidis*, *B. stearothermophilus*, *Staphylococcus typhimurium*, *Escherichia coli*, *P. aeruginosa* (32–11 mm)	[73]
	PP//HPLC	Black tea//5 min, 100 °C, 1 L W, 100 g sucrose, soy whey	*S. aureus*, *E. coli*, *Bacillus subtilis* (0.9–1.9 mm)	[52]
	PP//ESI-MS	Black tea//5 min, 100 °C, 1 L W, 50 g sugar	*E. coli*, *Vibrio cholerae*, *Shigella flexneri*, *Salmonella Typhimurium*, *S. aureus* (4.3–20.7 mm)	[47]
	PP, OA//TP, FTIR	Black tea//5 min, 100 °C, 1 L W, 30 g sugar	*Candida krusei*, *Candida glabrata*, *Candida albicans*, *Candida. tropicalis*, *Haemophilus influenzae*, *E. coli* (4–16 mm)	[15]
	OA//HPLC, TP	Green, oolong, and black tea//15 min, 100 °C, 1 L W, 100 g sucrose	*E. coli*, *Shigella dysenteriae*, *Salmonella Typhi*, *Vibrio cholera* (15–25 mm)	[81]
	Nd	Tea leaves//15 min, 100 °C, 1 L W with lemon balm, 70 g sucrose	*Pseudomonas aeruginosa*, *Proteus mirabilis*, *E. coli*, *Salmonella enteritidis*, *Erwinia carotovora*, *S. aureus*, *Bacillus cereus*, *Sarcina lutea* (13.67–17.83 mm)	[92]
	Nd	Green tea//15 min, 100 °C, 1 L W, 20 g sugar	*S. epidermidis*, *Listeria monocytogenes*, *Micrococcus luteus* (21.5–22.0 mm)	[95]
In vitro (MIC)	PP//TP	Green tea//5 min, 100 °C, 700 mL W, 35 g sucrose, *Malvaviscus arboreus*	*C. albicans*, *Cryptococcus neoformans*, *Paracoccidioides brasiliensis*, *S. aureus*, *L. monocytogenes*, *E. coli*	[75]
	PP//TP, HPLC	Green tea//15 min, 100 °C, 1 L W, 65 g sucrose, cinnamon, cardamom, or Shirazi thyme	↑ activity kombucha with cinnamon (*S. aureus*, *Bacillus cereus*, *E. coli*, *S. typhimurium*)	[78]
	PP//UHPLC-HRMS	Green tea//20 min, 100 °C, 1 L W, 100 g sucrose	*Alicycobacillus acidoterrestris*, *Alicyclobacillus herbarius*, *Alicyclobacillus acidiphilus*, *Alicyclobacillus cycloheptanicus*, *Alicyclobacillus hesperidum* (<50–12.5%)	[80]
	PP//ESI-MS	Black tea//5 min, 100 °C, 1 L W, 50 g sugar	*Vibrio cholerae* (800 μg/mL)	[96]
	Bacteriocin SLG10//HPLC	Commercial preparation	*B. subtilis*, *B. cereus*, *M. luteus*, *Brochothrix thermosphacta*, *Clostridium butyricum*, *S. aureus*, *Listeria innocua*, *L. monocytogenes*, *E. coli*, *P. aeruginosa*, *Enterobacter cloacae*, *Salmonella paratyphi* (nd-32 μg/mL)	[97]
In vitro (RSA)	PP//UPLC-QTOF-MS	Green and black tea//2–4 min, 75–95 °C, 1 L W, 50 g sucrose	↑ activity green tea (250 µL/mL)	[43]
	**Anti-inflammatory**			
In vitro (macrophages)	PP//LC-ESI-MS/MS	Black tea, oak//10 min, 80 °C, 1 L W, 100 g sucrose	Nitric oxide, TNF-alpha, and IL-6 production	[85]
	PP, OA//CG-MS	Black tea//15 min, 80 °C, 1 L W, 70 g sugar	28–44.5%	[68]
**Antidiabetic**				
In vivo (rats)	PP//TP	Black tea//10 min, 100 °C, 1 L W, 100 g sugar	↓ fasting plasma glucose; ↑ oxidative stress indices and lipid profiles	[98]
	PP//TP	Black tea//5 min, 100 °C, 1,6 L, 160 g sucrose	150 mg lyophilized extract/kg body weight 14 days; restored alloxan-induced pathophysiological changes	[87]
	Nd	Commercial preparation	6 mg/kg body weight 45 days; ↓ glycosylated hemoglobin; ↑ plasma insulin, hemoglobin, tissue glycogen; reversed the altered activities of gluconeogenic enzymes	[99]
	Nd	Commercial preparation	5 mL/kg body weight 30 days; inhibitor of α-amylase and lipase; suppressor of increased blood glucose levels	[100]
**Hypocholesterolemic**				
In vivo (rats)	PP//HPLC	Black tea//15 min, 100 °C, 1 L W, 100 g sucrose	↓ total cholesterol and LDL-cholesterol	[88]
In vivo (ducks)	Nd	Commercial preparation	↓ total cholesterol and LDL-cholesterol	[101]

Abbreviatures. Bioactive compounds. PP: polyphenols; OA: organic acids; Nd: not determined; Detection method. TP: total phenolic compounds determination using Folin–Ciocalteu method; HPLC: high-performance liquid chromatography; GC-MS: gas chromatography–mass spectrometry; LC-ESI-MS/MS: liquid chromatography–electrospray ionization–tandem mass spectrometry; UHPLC-HRMS: ultra-high-performance liquid chromatography–high-resolution mass spectrometry; UHPLC-QTOF-MS: ultra-high-performance liquid chromatography–quadrupole time-of-flight mass spectrometry; FTIR: Fourier transform infrared spectroscopy; NMR: nuclear magnetic resonance spectroscopy; ESI-MS: electrospray ionization-mass spectrometry; Infusion. W: water; Model and results. RSA: radical scavenging assays; ALX: alloxan monohydrate; ↑: enhanced; ↓: decrease; ACE: angiotensin-converting enzyme assay; Hep-2: human epithelial type 2; MCF7: Michigan cancer foundation-7; HCT-116: human colorectal carcinoma-116; A549: adenocarcinomic human alveolar basal epithelial cells; U2OS: human osteosarcoma; 786-O: human renal carcinoma; CACO-2: colon adenocarcinoma-2; OVCAR: ovarian carcinoma; HeLa: henrietta lacks; HT-29: human colon adenocarcinoma-29; PC-3: prostate cancer cell-line; DDM: disk diffusion method; MIC: minimum inhibitory concentration.

**Table 3:** Alternative kombucha formulations.

Secondary Fermentation	Primary Fermentation	BC	Results	Ref.
Lemon balm (5 g/L)	Black tea//15 min, 100 °C, 1 L W, 70 g sucrose//7 days//SCOBY Y: *Saccharomycodes ludwigii*, *Saccharomyces cerevisiae*, *Saccharomyces bisporus*, *Torulopsis* sp., *Zygosaccharomyces* sp./B: *Acetobacter genus*	RA, AA	TPC 850.3 µg GAE/L, AO, AM (*Listeria* spp., *Escherichia coli*, *Salmonella* sp., *Citrobacter freundii*, *Staphylococcus equorum*, *Staphylococcus saprophyticus*, *Bacillus* sp.)	[51]
Lemon balm	Tea leaves//15 min, 100 °C, 1 L W with lemon balm, 70 g sucrose//SCOBY Commercial preparation (nd)	Nd	AM (*Pseudomonas aeruginosa*, *Proteus mirabilis*, *E. coli*, *Salmonella enteritidis*, *Erwinia carotovora*, *Staphylococcus aureus*, *Bacillus cereus*, *Sarcina lutea*), AT	[92]
Arabica coffee (2–13%)	Green tea//6 min, 93 °C, 1 L W//18 days//SCOBY Commercial preparation (nd)	GA	↓ luminosity; low-calorie; ↑ consumer acceptation	[126]
Cocoa mucilage	Green tea//up to 100 g/L sugar//15 days//SCOBY Commercial preparation (nd)	PP	↑ consumer acceptation	[127]
Oak (1%)	Black tea//10 min, 80 °C, 1 L W, 100 g sucrose//7 days//SCOBY Commercial preparation (nd)	PP	AI	[85,128]
Yarrow (2%)	Black tea//15 min, 100 °C, 1 L W//7 days//SCOBY Y: *S. ludwigii*, *S. cerevisiae*, *S. bisporus*, *Torulopsis* sp., *Zygosaccharomyces* sp./B: *Acetobacter genus*	AA, OA, FA, SA, MA, CA	AM, CY, ↑ consumer acceptation	[129]
Wax mallow (7%)	Green tea//5 min, 100 °C, 700 mL W, 35 g sugar//18 days//SCOBY *Medusomyces gisevii*	AA	TPC 124.6 mg GAE/L, AO	[75]
Sour cherry juice	Green tea//5 min, 100 °C, 1 L W, 80 g sugar//14 days//SCOBY Osmophilic Y, acetic acid bacteria	GA	AO	[130]
Cactus pear juice (10%)	Black tea//5 min, 100 °C, 400 mL W, 40 g saccharose//15 days//SCOBY nd	GU	TPC 857 mg GAE/L, AO, AM (*S. aureus*, *Staphylococcus epidermidis*, *Enterococcus faecalis*, *B. cereus*, *Klebsiella pneumoniae*, *Pseudomonas aeruginosa*, *E. coli*)	[131]
Snake fruit juice	Black tea//10 min, 100 °C, 1 L W, 100 g sugar//15 days//SCOBY nd	PP	TPC 535.59 mg GAE/L, AO, AD	[98]
Soy milk (1:2 *v*/*v*)	Black tea//10 min, 28 °C, 1 L soymilk, 70 g sucrose//10 days//SCOBY *Acetobacter*, *Lactobacillus*	AA, GA, MA, CA, LA	TPC 3.58 GAE/g dw, AO	[82]
Soy whey (1:10 *v*/*v*)	Black tea//5 min, 100 °C, 1 L W, 100 g sucrose//7 days//SCOBY Y: *Pichia*, *Dekkera/*B: *Acetobacter*, *Lactobacillus*	PP	AO (EC50 1.66–9.11 mg extract/mL), AM (*S. aureus*, *E. coli*, *Bacillus subtilis*)	[52]
Cinnamon (50:50 *w*/*w*)	Green tea//15 min, 100 °C, 1 L W, 65 g sucrose//16 days//SCOBY Commercial preparation (nd)	AA, GA, CA, LA	TPC 0.55 mg GAE/mL, AO, AM (*S. aureus*, *B. cereus*, *E. coli*, *Salmonella typhimurium*, *Helicobacter pylori*)	[78]
Cardamon (50:50 *w*/*w*)	Green tea//15 min, 100 °C, 1 L W, 65 g sucrose//16 days//SCOBY Commercial preparation (nd)	AA, GA, CA, LA	TPC 0.45 mg GAE/mL, AO, AM (*S. aureus*, *B. cereus*, *E. coli*, *S. typhimurium*)	[78]
Mint (10 g/L)	Black tea//100 °C, 1 L W, 50 g sucrose//13 days//SCOBY Commercial preparation (nd)	AC	↑ consumer acceptation	[132]
Ginger	Black tea//100 °C, 1 L W, 10 g sucrose//10 days//SCOBY Commercial preparation (nd)	nd	AO, AT	[133]
Papaya (10% *w*/*v*)	1 L W, 10 g sugar//4 days//SCOBY Commercial preparation (nd)	AA	Further optimization needed	[134]
Papaya (10 g/L)	20 min, 1 L W, 100 g sugar//14 days//SCOBY Y: *Brettanomyces bruxellensis*, *S. cerevisiae*/B: *Komagataeibacter pomaceri*, *Komagataeibacter rhaeticus*	PP	AM (*S. aureus*, *Salmonella typhi*, *E. coli*), AD, ↑ consumer acceptation	[135]
Pomegranate juice	Black tea//100 °C, 5 min, 200 mL W, 8 g sucrose//14 days//SCOBY Y: Saccharomyces, Koleckera, Pichia, Schizosaccharomyces/B: *Acteobacter* spp., *Gluconobacter* spp.	GA	Potential functional beverage candidate due to GA content	[136]
Red grape juice	Black tea//100 °C, 5 min, 200 mL W, 20 g saccharose//15 days//SCOBY Y: Zygosaccharomyces, Saccharomyces/B: *Acetobacter xylinus*, *Acetobacter pasteurianus*, *Gluconobacter oxydans*	PP	AO, AM, ↑ consumer acceptation	[137]
Grape by-products (20% *v*/*v*)	Green tea//15 min, 90 °C, 1 L W, 30 g sucrose//15 days//SCOBY Commercial preparation (nd)	PP	TPC 227.34 mg GAE/L	[138]
Mango by-products (20% *v*/*v*)	Green tea//15 min, 90 °C, 1 L W, 30 g sucrose//15 days//SCOBY Commercial preparation (nd)	PP	TPC 165.77 mg GAE/L	[138]
Banana peel (10 g)	Green tea//5 min, 100 °C, 1 L W, 20 g sucrose//21 days//SCOBY Commercial preparation (nd)	PP, AC	TPC 530.5 ppm, AO	[139]
Flavor additives	Green tea-based kombucha and different additives (lemongrass; lavender; liquorice-mint; turmeric-lemon; mango; reishi-chaga; mint-rose-pomegranate)	PP	TPC up to 265.82 mg GAE/L, AO	[140]

Abbreviatures. Bioactive compounds. BC: bioactive compounds; RA: rosmarinic acid; AA: acetic acid; GA: glucuronic acid; PP: polyphenols; OA: oxalic acid; FA: formic acid; SA: succinic acid; MA: malic acid; CA: citric acid; GU: gluconic acid; LA: lactic acid; CA: caffeine; AC: organic acids; SCOBY. Y: yeast; B: bacteria. Results. TPC: total phenolic content; AO: antioxidant; AM: antimicrobial; AT: anticancer; AI: anti-inflammatory; CY: cytotoxic; AD: antidiabetic; ↑: enhanced; ↓: decrease.

## Data Availability

No new data were created or analyzed in this study. Data sharing is not applicable to this article.
